# Research on comorbidity characteristics and patterns of hospitalized participants with schizophrenia in China

**DOI:** 10.3389/fpsyt.2025.1619051

**Published:** 2025-07-25

**Authors:** Xinru Huang, Ting Zhang, Bin Li, Yahui Meng, Fen Li

**Affiliations:** ^1^ School of Management, Xuzhou Medical University, Xuzhou, Jiangsu, China; ^2^ Medical Affairs Department, Huai’an No.3 People’s Hospital, Hui’an, China; ^3^ Emergency Department, Xuzhou Oriental People’s Hospital, Xuzhou, Jiangsu, China; ^4^ Medical Affairs Department, Xuzhou Cancer Hospital, Xuzhou, Jiangsu, China

**Keywords:** schizophrenia, comorbidity characteristics, comorbidity mode, association rule analysis, hospitalized participants

## Abstract

**Introduction:**

Participants with schizophrenia face the dual impacts of mental illness and physical diseases, which significantly affect their clinical prognosis and quality of life.

**Methods:**

This study, which is based on multivariate clinical data, using data-driven approaches (statistical analysis and data mining techniques) to systematically characterize the comorbidities landscape of schizophrenia patients identifying key patterns and clinical profiles to inform optimized treatment strategies and health management interventions.

**Results:**

It was found that the comorbidity rate among hospitalized schizophrenia participants is notably high in China. Notably, significant variations in comorbidity profiles were identified across diverse demographic and clinical variables, including age, occupational status, marital status, duration of hospital stay, frequency of hospitalizations, and health insurance status. The comorbidities in schizophrenia participants primarily include acute upper respiratory infections (J00-J06), metabolic disorders (E70-E90), hypertension (I10-I15), and diabetes (E1 0-E14). Furthermore, four strongly associated comorbidities patterns were identified: the metabolic-cardiovascular comorbidities cluster, the co-disease cluster of respiratory system infection, gut respiratory symptom cluster, electrolyte imbalance infection trigger cluster were identified.

**Discussion:**

The exploration of comorbidity characteristics and patterns in schizophrenia provides a quantifiable tool for enhancing treatment and health management outcomes for participants while also offering a reference for advancing the application of precision medicine in the treatment and management of schizophrenia.

## 1 Literature review and problem statement

As a crucial component of overall well-being, mental health represents a significant public health and social issue that impacts socioeconomic development and the advancement of healthy ageing. The influence of severe mental disorders on healthy life expectancy is particularly profound. Severe mental disorders are characterized by prolonged and fluctuating courses, high recurrence rates, poor prognoses, elevated disability rates, and substantial economic burdens on families (1–4). Severe mental disorders not only impose heavy treatment, rehabilitation, and caregiving burdens on individuals and their families but also pose considerable challenges to social security and governance.

### 1.1 Comorbidity patterns increase health risks in schizophrenia patients

The emerging concept of comorbidity is predominantly used in chronic disease research and refers to the coexistence of two or more chronic conditions (5, 6). It is a patient-centered framework aimed at conceptualizing the number and types of diseases. The study of comorbidity patterns has gradually expanded beyond chronic diseases to other disease areas. In mental health research, exploring comorbidity patterns has garnered increasing attention. Studies have revealed that 31% of participants with schizophrenia suffer from multiple complex conditions, commonly including combinations of endocrine diseases, respiratory diseases, epilepsy, and arthritis (7, 8). Additionally, hypertension, diabetes, and asthma are also frequently observed as comorbid conditions (9). Moreover, the co-occurrence of multiple mental disorders is relatively high, 14% have psychiatric multimorbidity (10).

The mortality rate among participants with severe mental disorders is often attributed to the presence of comorbid conditions, which increase health risks and consequently lead to higher mortality rates. Severe mental disorders are also major risk factors for disability among participants (11). Research indicates that patients with schizophrenia had a higher risk of death associated with COVID-19 infection. Nevertheless, they demonstrated a good immune response to vaccination, which highlights the importance of immunization for this vulnerable group (12, 13). Studies have shown that 40% of participants with severe mental disorders suffer from two or more comorbid conditions, with 20% of participants having two or more physical illnesses in addition to severe mental disorders. Furthermore, two-thirds of deaths among participants with severe mental disorders are caused by increased risks associated with comorbidities (14–18).

### 1.2 Health impacts and multidimensional challenges of comorbidity patterns in schizophrenia

Schizophrenia, a severe mental disorder with a relatively high incidence rate, is often compounded by unhealthy behaviors and lifestyle factors that contribute to the development of somatic comorbidities (18). Specifically, the comorbidity of schizophrenia with alcohol and substance use disorders is particularly concerning. These comorbid conditions not only increase the overall disease burden but also significantly exacerbate the risk and severity of physical comorbidities. Moreover, individuals with schizophrenia who are dependent on drugs tend to experience poorer quality of life (19, 20).

Schizophrenia significantly impact the life expectancy of participants (21), exerting profound effects on both their physical and psychological well-being (22). Research on comorbidity patterns in individuals with mental disorders has focused primarily on cardiometabolic risk factors, immune system diseases, and the factors influencing comorbidity patterns (23, 24). With the evolution of the “New Health” concept, disease treatment has gradually shifted from a single-disease treatment model to comprehensive treatment and intervention. The increasing prevalence of comorbidities has amplified the pressure on caregiving and the economic burden associated with diseases. It also impacts the accumulation of health capital over a lifetime, thereby becoming a detrimental driver of health outcomes (25). In current research on comorbidity patterns, the primary approach involves using the International Classification of Diseases (ICD-10) as the basis for disease categorization. This method analyses the associations between different disease categories in participants to uncover patterns of disease co-occurrence.

Research on comorbidity patterns in schizophrenia remains relatively limited. Compared with general diseases, schizophrenia encompass both physiological and psychological conditions, resulting in particularly complex comorbidity patterns. Investigating these patterns is crucial for providing comprehensive treatment and intervention for participants. This study focused on participants with schizophrenia, a type of severe mental disorder, with the aim of exploring and analyzing the disease characteristics, comorbidity features, and comorbidity patterns of these participants.

## 2 Data sources and methods

### 2.1 Data sources

The “Management Measures for Reporting the Incidence of Severe Mental Disorders (Trial)” (National Health and Family Planning Commission [2013] No. 8) in China defines severe mental disorders as mental disorders with severe symptoms that cause serious impairment of participants’ social adaptation and other functions, inability to fully understand their own health status or objective reality, or inability to handle their affairs. These include six serious mental disorders: schizophrenia, schizoaffective disorder, paranoid psychosis, bipolar disorder, epilepsy-induced mental disorders, and intellectual disability accompanied by mental disorders, and participants who meet the requirements of Article 30, Paragraph 2, and Item 2 of the Mental Health Law of the People’s Republic of China and have been diagnosed and assessed as having serious mental disorders. This study used the category codes corresponding to the International Classification of Diseases (ICD) as the classification criteria and selected samples with category code F20 from electronic medical records as the screening criteria for participants with schizophrenia.

This study was conducted in three psychiatric speciality hospitals in Jiangsu Province, China. Based on the practical circumstances of the hospital’s electronic medical record (EMR) system and hospital information system (HIS). And to protect the requirements of scientific research and patient privacy, we deleted personally identifiable information (PII) including names, government IDs, and phone numbers during the data export phase, and generalized residential addresses to regional codes. We exported and collected a total of 18, 622 inpatient medical records from January 1, 2023, to June 30, 2024, including 4, 596 cases of participants with schizophrenia. To ensure data quality, we implemented validation measures, including cross-checking between birth dates and ages and verifying disease names against ICD-10 codes. For entries with identified discrepancies, we collaborated with hospital medical record quality control staff to review and correct the data to enhance its accuracy and standardization.

### 2.2 Research methods

#### 2.2.1 General statistical methods

The database was constructed by exporting Excel data from the hospital’s EMR and HIS system. Data processing and analysis were performed using Stata software to examine the disease characteristics, comorbidity patterns, and influencing factors of schizophrenia participants. The ethical approval number for this study is 20231028002.

The statistics of comorbidities are based on the main diagnosis codes and names in electronic medical records as the main disease categories and the diagnosis codes and names of secondary diseases as the selection criteria for comorbidities. The electronic medical records included diagnostic data for 10 types of secondary diseases. In the data processing stage, for the statistics of the number of secondary disease diagnoses, with “Secondary Disease Diagnosis 1” as an example, if the secondary diagnosis includes a certain disease, it is assigned a value of 1, if the secondary disease diagnosis does not include a certain disease, it is assigned a value of 0, and the sum of secondary disease diagnoses 1–10 is added.

To further analyze the differences in comorbidities among hospitalized patients with schizophrenia, the number of comorbidities was used as the dependent variable, and the independent variables were gender, age, occupational status, marital status, primary contact person, hospitalization days, times of hospitalizations, and Medical insurance. Due to the range of values for the number of comorbidities being 0-10, which is a non-negative count variable, Poisson Multiple Regression analysis is preferred based on the type of dependent variable. However, when testing the discreteness of the dependent variable, it was found that the variance of the dependent variable ( φ=1.604, φ>1 ) was significantly greater than expected, indicating overdispersion. Therefore, this study used Negative Binomial Regression to explore the differences in comorbidities among hospitalized patients with schizophrenia.

#### 2.2.2 Association rule analysis

Association rule analysis is commonly used in data mining, aiming to uncover hidden relationships and the degree of association between data items by identifying patterns in two or more variables (26). Association rules are used to evaluate the strength of these relationships through metrics such as support, confidence, and lift. These measures are employed to explore comorbidity patterns in participants with schizophrenia.

Support refers to the probability of the itemsets {A, B} appearing in the sample, that is, the probability of both A and B appearing simultaneously in the itemsets, as shown in Equation (1). By setting a minimum threshold (minsup) for support, the itemsets with higher support are retained. The calculation formula for support is as follows:


(1) Support(A>B)={A, B}diseases/total number of hospitalizations

Confidence refers to the probability that a patient has disease B under the condition of having disease A, as shown in Equation (2). Similarly, we can set a minimum threshold for confidence (mincon), and the formula for calculating confidence is as follows:


(2) Confidence(A−>B)= {A, B} diseases/number of people with disease A

Lift refers to the ratio of the likelihood of a patient having both disease A and disease B to the likelihood of not having disease A but having disease B, as shown in Equation (3). If the degree of lift is equal to 1, it indicates that there is no correlation between disease A and disease B. If the degree of lift is less than 1, it indicates that disease A and disease B are mutually exclusive. If the degree of lift is greater than 1, it indicates a correlation between disease A and disease B. The formula for calculating the degree of lift of disease A on disease B is as follows:


(3) Lift(A−>B)=({A, B} diseases/number of people with disease A)/(disease B/total number of hospitalizations)

This study adopts the method of association rule analysis, using support, confidence, and lift to explain the strength of comorbidity associations in participants with schizophrenia. This study set minimum thresholds for support, confidence, and lift. The minimum threshold for support was 1.5%, the minimum threshold for confidence was 30%, and the minimum threshold for lift was 1. That is, when comorbidities that account for 1.5% or more of the total survey population in the sample are selected, the probability of having one disease while having another disease is greater than or equal to 30%, and the likelihood of having B disease while having A disease is greater than the likelihood of not having A disease but having B disease. This method was utilized as an association rule to unearth comorbidity patterns among participants diagnosed with schizophrenia.

## 3 Results

### 3.1 Analysis of basic characteristics of the sample population

As shown in **Table 1**, the number of male participants with schizophrenia was greater than female participants. In terms of age distribution, participants had a mean age of 42.042 years (SD=14.257), the proportion of people aged 30–69 years is 69.84%, to better distinguish comorbidities differences across age groups, participants were stratified into six age categories. Among participants with schizophrenia, the proportion of unemployed individuals was the highest, accounting for 61.34%, and due to the nature of the disease, participants with schizophrenia are limited in employment compared with the general population. From the perspective of marital status, 44.69% of participants were unmarried, 36.60% were married, 16.14% were divorced, and 2.57% were widowed. Among hospitalized participants, the main contacts were parents and spouses. The average length of hospital stay per visit was 68.84 ± 40.61 days, and the average hospitalization frequency was 7.81 ± 6.71. A total of 77.05% of participants used resident medical insurance for medical expense settlement. Participants with schizophrenia are characterized by a long disease course and easy recurrence, which to some extent poses significant challenges to the payment of medical insurance and the disease burden.

**Table 1:** Demographic characteristics and values of participants with schizophrenia.

Variable	Variable assignment	Percentage/Mean
Sex	1=female	42.62
	2=male	57.38
Age	1=Under 18 years old	2.22
	2 = 18-29	18.86
	3 = 30-44	30.18
	4 = 45-59	39.66
	5 = 60-69	7.09
	6 = 70 years old and above	1.98
Occupation	1= unemployed	61.34
	2= farmer	17.04
	3= worker	8.88
	4= student	3.31
	5= retiree	6.64
	6= other	2.81
Marital Status	1= unmarried	44.69
	2= married	36.60
	3= widowed	2.57
	4= divorced	16.14
Contacts	1= parents	45.49
	2= spouses	18.68
	3= children	4.90
	4= Brothers or sisters	3.31
	5=other	27.62
Hospitalization days	Unit: Day	68.84 ± 40.61
Hospitalization times	Unit: times	7.81 ± 6.71
Medical insurance	1= resident medical insurance	77.05
	2= employee medical insurance	15.40
	3= pay out of pocket	7.25
	4=other	0.30

### 3.2 Analysis of comorbidity characteristics in hospitalized participants with schizophrenia

#### 3.2.1 Comorbidity and age distribution of participants with schizophrenia

Therefore, the number of secondary diseases diagnosed by participants in addition to the primary diagnosis is called the “comorbidity count”, which ranges from 0 to 10. Statistical analysis revealed that the comorbidity rate of hospitalized participants with schizophrenia was 72.63%, as shown in **Table 2**.

**Table 2:** Statistics of comorbidities among hospitalized participants with psychiatric disorders.

Age Counts	<18	18-29	30-44	45-59	60-69	≥70	Samples	(%)
0	40	314	449	371	14	2	1190	25.89
1	35	293	418	461	71	11	1289	28.05
2	19	178	304	405	61	18	985	21.43
3	4	60	135	302	82	20	603	13.12
4	4	11	47	152	43	12	269	5.85
5	0	6	20	74	27	10	137	2.98
6	0	5	8	27	11	5	56	1.22
7	0	0	2	13	8	4	27	0.59
8	0	0	2	10	6	1	19	0.41
9	0	0	1	7	3	5	16	0.35
10	0	0	1	1	0	3	5	0.11
Total	102	867	1387	1823	326	91	4596	100.00

In terms of age distribution, the comorbidity rate of among hospitalized participants with schizophrenia varied across age groups: Under 18 years of age, the comorbidity rate was 60.78%, 18–29 years of age, the comorbidity rate was 63.78%, 30–44 years of age, the comorbidity rate was 67.63%, 45–59 years of age, the comorbidity rate was 79.65%, 60–69 years of age, the comorbidity rate was 95.71%, and 70 years or older, the comorbidity rate was 97.80%.

From **Table 3**, schizophrenia participants aged<18 years had the lowest comorbidity burden (median=0, IQR:0-1), indicating that 50% of participants had 0 to 1 comorbidity. The 18–29 and 30–44 year groups both showed median=1 comorbidity (IQR:0-2), meaning half of participants presented with 0 to 2 comorbidities. Middle-aged participants (45–59 years) exhibited median=2 comorbidities (IQR:1-3), indicating that 50% of participants had 1 to 3 comorbidities. While those aged 60–69 years showed increased burden (median=3, IQR:1-4), indicating that 50% of participants had 1 to 4 comorbidities. The oldest cohort (≥70 years) had the highest comorbidity load (median=3, IQR:2-5), indicating that 50% of participants had 2 to 5 comorbidities. As age increased, both the comorbidity rate and average number of comorbidities among hospitalized participants with schizophrenia progressively increased.

**Table 3:** Comorbidities in hospitalized schizophrenia participants by age group: described using quartiles.

Age	Q1	Median	Q3	IQR
<18	0	0	1	1
18-29	0	1	2	2
30-44	0	1	2	2
45-59	1	2	3	2
60-69	1	3	4	3
≥70	2	3	5	3

Q1 = 25th percentile, Q3 = 75th percentile, IQR=interquartile range.

#### 3.2.2 Differential analysis of comorbidities in participants with schizophrenia

As shown in **Table 4**, the analysis of Negative Binomial Regression revealed no significant sex differences among hospitalized participants with schizophrenia. In terms of age groups, a positive correlation was observed between age and the number of comorbidities. In the control group(<18years), participants aged 18–29 years and 30–44 years presented no significant difference in the number of comorbidities compared with the control group. Participants aged 45–59 years had, on average, 0.254 more comorbidities than participants in the control group (<18years), adjusting for other variables. Participants aged 60–69 years had, on average, 0.666 more comorbidities than participants in the control group (<18years), adjusting for other variables. Participants aged70 years or older had, on average, 0.893 more comorbidities than participants in the control group (<18years), adjusting for other variables.

**Table 4:** Statistical results of robustness analysis on comorbidity differences among hospitalized participants with schizophrenia.

Variable		Coefficient	Robust std.err.	p>|z|	[95%conf. interval]
Sex (female)					
male		-0.009	0.029	0.755	-0.067, 0.048
Age (<18)					
18-29		-0.115	0.112	0.307	-0.335, 0.105
30-44		-0.032	0.115	0.780	-0.258, 0.193
45-59		0.254	0.117	0.029	0.026, 0.483
60-69		0.666	0.121	0.000	0.428, 0.905
≥70		0.893	0.134	0.000	0.631, 1.156
Occupation (Unemployed)					
farmer		-0.007	0.037	0.850	-0.079, 0.065
worker		0.013	0.047	0.786	-0.080, 0.105
student		-0.349	0.118	0.003	-0.580, -0.118
retiree		0.048	0.060	0.421	-0.069, 0.166
other		-0.174	0.100	-0.081	-0.370, 0.021
Marital Status (unmarried)					
married		0.105	0.038	0.005	0.031, 0.179
widowed		0.013	0.075	0.868	-0.135, 0.160
divorced		0.069	0.040	0.086	-0.024, 0.257
Contacts (parents)					
spouses		-0.022	0.043	0.606	-0.107, 0.062
children		-0.064	0.059	0.274	-0.180, 0.051
brothers or sisters		0.001	0.077	0.991	-0.150, 0.152
other		0.053	0.034	0.119	-0.013, 0.119
Hospitalization days		0.003	0.001	0.000	0.003, 0.004
Times of hospitalizations		0.007	0.002	0.003	0.002, 0.011
Medical insurance (resident)					
employee		0.035	0.043	0.041	-0.049, 0.120
self-paid		-0.193	0.065	0.003	-0.320, -0.028
other		0.267	0.207	0.197	-0.139, 0.673
Constant		0.001	0.121	0.997	-0.237.0.238
lnalpha		-1.949	0.115	–	-2.175, -1.723
alpha		0.142	0.016	–	0.114, 0.179
	Samples=4594				
	Prob>chi2 = 0.000				
	Pseddo R2 = 0.052				

1. The parentheses after the independent variable indicate the control group.

For occupational status, with unemployed individuals as the control group, there was no significant difference in the number of comorbidities between farmers, workers, and retired individuals and the control group (Unemployed). The number of comorbidities in the student group and other groups was lower than that of unemployed individuals. From the perspective of marital status, when unmarried individuals were used as the control group, there was no significant difference in the number of cases between the widowed and divorced groups and the control group. There was a significant difference in the number of comorbidities between the married and unmarried groups, and married schizophrenia participants had a greater number of comorbidities than unmarried participants.

The primary contact of hospitalized participants had no significant effect on the number of comorbidities. There was a significant positive correlation between the number of comorbidities in hospitalized participants with schizophrenia and the duration and frequency of a single hospitalization. For every additional day of hospitalization, the number of comorbidities in participants increased by 0.003. The greater the number of comorbidities in participants with schizophrenia, the longer the length of hospital stay and the greater the number of hospitalizations. For medical insurance type, when participants with urban-rural resident medical insurance were used as the control group, self-paid participants presented a lower likelihood of comorbidities. This result may stem from adverse selection in the insurance enrolment process, where individuals with preexisting or multiple health conditions were more inclined to purchase medical insurance to secure health protection.

### 3.3 Analysis of comorbidity patterns in participants with schizophrenia

**Figure 1:**
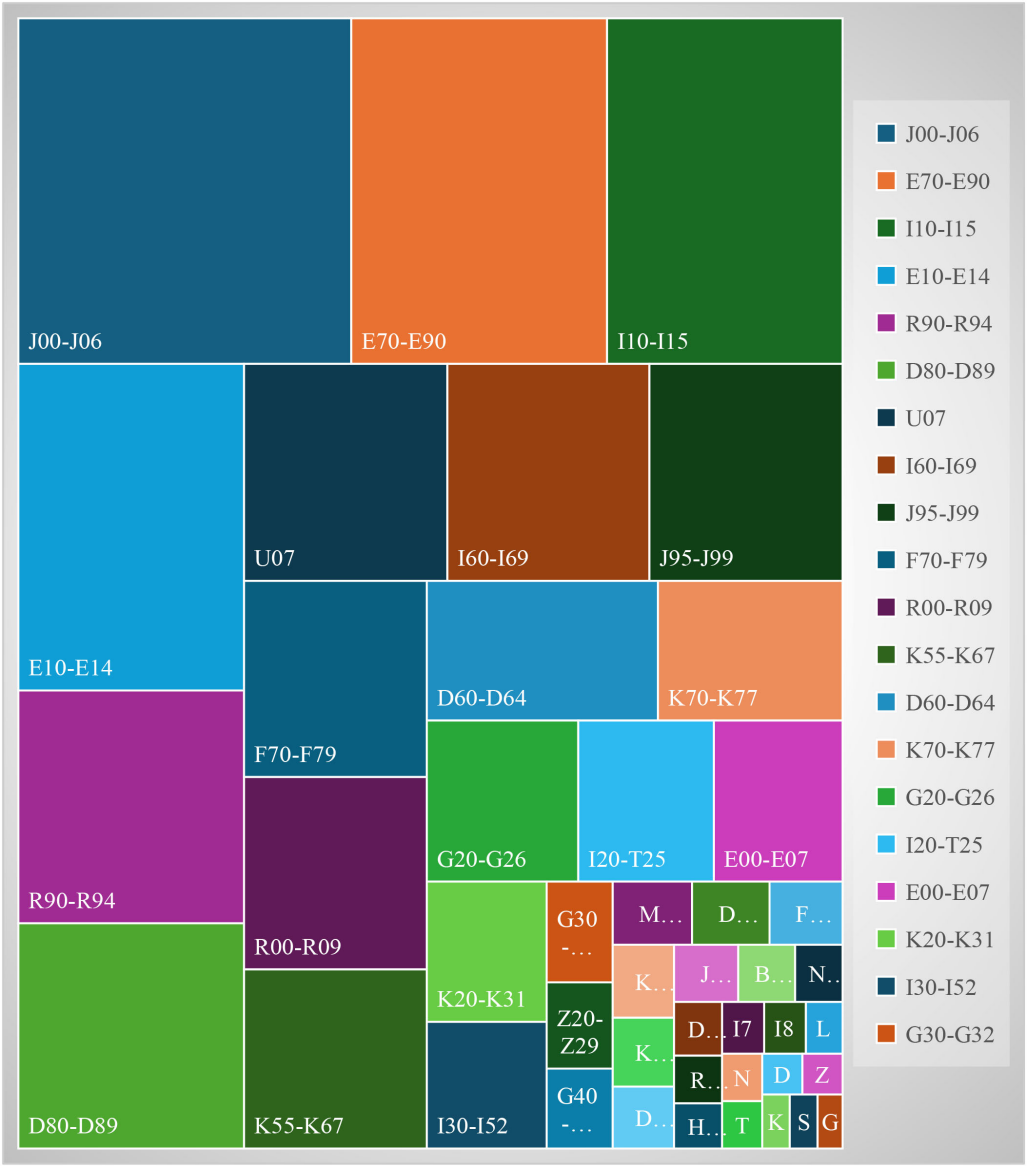
Comorbidity distribution of participants with schizophrenia (F20) under different ICD-10 category coding classifications.

According to the classification code analysis of the ICD-10, among participants with schizophrenia (F20), by analyzing the distribution of comorbidities and ranking them according to the prevalence rate from high to low, the top 10 disease groups are acute upper respiratory tract infection (J00-J06), metabolic disorders (E70-E90), hypertension (I10-I15), diabetes (E10-E14), abnormal findings of diagnostic impact and functional examination (R90-R94), some diseases involving immune mechanisms (D80-D89), COVID-19 infection (U07), cerebrovascular disease (I60-I69), other diseases of the respiratory system (J80-D89). 95-J99), and intellectual disability (F70-F79), as shown in **Figure 1**.

According to the ICD-10 code, the top 10 diseases of participants with schizophrenia in terms of the number of comorbidities ordered from most to least common: acute nasopharyngitis (J00. x00), hypokalaemia (E87.600), type 2 diabetes (E11.900), leukopenia (D70. x04), liver dysfunction (R94.500), COVID-19 infection (U07.100x002), lung infection (J98.414), constipation (K59.000), grade 3 hypertension (extremely high risk) (I10. x00.032), and lacunar cerebral infarction (I63.801).

### 3.4 Mining comorbidity patterns in hospitalized participants with schizophrenia

An analysis of comorbidity characteristics in participants with schizophrenia revealed that the comorbidity rate was high and there are many types of comorbidities. However, further exploration is needed to determine association patterns and strengths between various diseases and schizophrenia. Only with a clear association pattern can the treatment efficiency of participants with schizophrenia and the exploration of patient care models be improved.

#### 3.4.1 Analysis of comorbidity support for participants with schizophrenia

Through association rule analysis, it was found that among participants with schizophrenia, there are nine association rules in terms of support. Among them, those with the highest support rates were {hypokalaemia ->acute nasopharyngitis [cold]} and {acute nasopharyngitis [cold]->hypokalaemia}, both with support rates of 2.35%. As shown in **Table 5**, the rules{hypokalaemia, schizophrenia ->acute nasopharyngitis[cold]}, {schizophrenia, acute nasopharyngitis[cold] ->hypokalaemia}, {hypokalaemia ->schizophrenia, acute nasopharyngitis [cold]}, and {acute nasopharyngitis[cold] ->hypokalaemia, schizophrenia} all shared the same support of 2.35%. These findings indicate that the proportion of schizophrenia participants with both hypokalaemia and acute nasopharyngitis [cold] was 2.35%.

**Table 5:** Analysis results of disease association rules for participants with schizophrenia.

Serials	A	B	Support	Confidence	Lift
Comorbidity Mode 1	Grade 3 hypertension (extremely high risk)	Type 2 diabetes	0.017	0.371	3.361
	Type 2 diabetes	Grade 3 hypertension (extremely high risk)	0.017	0.150	3.361
	Grade 3 hypertension (extremely high risk)	Type 2 diabetes	0.017	0.371	3.361
	Type 2 diabetes, schizophrenia	Grade 3 hypertension (extremely high risk)	0.017	0.151	3.374
	Grade 3 hypertension (extremely high risk)	Type 2 diabetes, schizophrenia	0.017	0.371	3.361
	type 2 diabetes	Grade 3 hypertension (extremely high risk)	0.017	0.150	3.361
Comorbidity Mode 2	Pulmonary infection	COVID-19 infection	0.017	0.250	3.514
	COVID-19 infection	Pulmonary infection	0.017	0.235	3.524
	Pulmonary infection, schizophrenia	COVID-19 infection	0.017	0.250	3.514
	Schizophrenia, COVID-19 infection	Pulmonary infection	0.017	0.233	3.479
	Pulmonary infection	Schizophrenia, COVID-19 infection	0.017	0.247	3.479
	COVID-19 infection	Pulmonary infection, schizophrenia	0.017	0.232	3.514
Comorbidity Mode 3	Constipation	Acute Nasopharyngitis [Cold]	0.015	0.290	1.779
	Schizophrenia, Constipation	Acute Nasopharyngitis [Cold]	0.015	0.290	1.779
	Constipation	Schizophrenia, Acute Nasopharyngitis [Cold]	0.015	0.290	1.779
Comorbidity Mode 4	Hypokalaemia	Acute Nasopharyngitis [Cold]	0.024	0.215	1.318
	Acute Nasopharyngitis [Cold]	Hypokalaemia	0.024	0.144	1.318
	Hypokalaemia, schizophrenia	Acute Nasopharyngitis [Cold]	0.024	0.216	1.328
	Schizophrenia, Acute Nasopharyngitis [Cold]	Hypokalaemia	0.024	0.144	1.318
	Hypokalaemia	Schizophrenia, Acute Nasopharyngitis [Cold]	0.024	0.215	1.318
	Acute Nasopharyngitis [Cold]	Hypokalaemia, schizophrenia	0.024	0.144	1.328

From the perspective of the comorbidity of schizophrenia, grade 3 hypertension (extremely high risk) and type 2 diabetes, {grade 3 hypertension (extremely high risk) ->type 2 diabetes}, {type 2 diabetes->grade 3 hypertension (extremely high risk), {grade 3 hypertension (extremely high risk), schizophrenia->type 2 diabetes}, {type 2 diabetes, schizophrenia->grade 3 hypertension (extremely high risk), {grade 3 hypertension (extremely high risk)->type 2 diabetes, schizophrenia}, and {type 2 diabetes ->grade 3 hypertension (extremely high risk), schizophrenia}. The support of these six comorbidity combinations was 1.65%; that is, among participants with schizophrenia, the proportion of participants with grade 3 hypertension (extremely high risk) and type 2 diabetes was 1.65%.

In terms of the comorbidity combination of schizophrenia, constipation, and acute nasopharyngitis[cold], the support rates for {constipation->acute nasopharyngitis [cold]}, {schizophrenia, constipation->acute nasopharyngitis[cold]}, and {constipation ->schizophrenia, acute nasopharyngitis[cold]} were all 1.5%. This means that among hospitalized participants with schizophrenia, 1.5% with constipation suffered from acute nasopharyngitis[cold].

For the combination of schizophrenia, pulmonary infection and COVID-19 infection, {pulmonary infection->COVID-19 infection} and {COVID-19 infection ->pulmonary infection} had a support of 1.68%, and {pulmonary infection, schizophrenia ->COVID-19 infection}, {schizophrenia, COVID-19 infection ->pulmonary infection}, {pulmonary infection ->COVID-19 infection, schizophrenia}, and {schizophrenia, COVID-19 infection ->pulmonary infection} had a support of 1.65%.

#### 3.4.2 Confidence analysis of comorbidities in participants with schizophrenia

For confidence level, the combination of comorbidities with the highest confidence level was {grade 3 hypertension (extremely high risk) -> type 2 diabetes}, {grade 3 hypertension (extremely high risk), schizophrenia -> type 2 diabetes}, and {grade 3 hypertension (extremely high risk) -> type 2 diabetes, schizophrenia}, and the confidence level was 37.07%; that is, the probability of type 2 diabetes among participants with schizophrenia and grade 3 hypertension (extremely high risk) was 37.7%. However, for schizophrenia participants with type 2 diabetes, the probability of having grade 3 hypertension (extremely high risk) was approximately 15%.

As shown in **Table 5**, the confidence levels for comorbidity combinations of {Constipation->Acute Nasopharyngitis [Cold]}, {Schizophrenia, Constipation ->Acute Nasopharyngitis[Cold]}, {Constipation->Schizophrenia, Acute Nasopharyngitis [Cold]}, and {Constipation ->Schizophrenia, Acute Nasopharyngitis [Cold]} were 28.99%. Therefore, the probability of schizophrenia participants with constipation suffering from acute nasopharyngitis [Cold] was 28.99%.

For the comorbidity patterns of schizophrenia, hypokalaemia, and acute nasopharyngitis [cold], the probability of participants with schizophrenia suffering from acute nasopharyngitis [cold] while suffering from hypokalaemia was 21.64%, whereas the probability of suffering from hypokalaemia among schizophrenia participants suffering from acute nasopharyngitis [cold] was 14.42%. This means that the probability of schizophrenia participants with hypokalaemia coexisting with acute nasopharyngitis [cold] was relatively high.

From the analysis of comorbidity patterns of schizophrenia, pulmonary infection and COVID-19 infection, the confidence level of {pulmonary infection->COVID-19 infection} was 25.00%, the confidence level of {COVID-19 infection->pulmonary infection} was 23.54%, and the confidence levels of comorbidity serial numbers {pulmonary infection, schizophrenia->COVID-19 infection}, {schizophrenia, COVID-19 infection->pulmonary infection}, {pulmonary infection->schizophrenia, COVID-19 infection}, and {COVID-19 infection->pulmonary infection, schizophrenia} were 25.00%, 23.31%, 24.68%, and 23.24%, respectively. Therefore, confidence in COVID-19 infection was relatively high in schizophrenia participants with lung infection.

#### 3.4.3 Analysis of the lift of comorbidity patterns in participants with schizophrenia

Based on the analysis of the support and confidence of the disease comorbidity patterns, to further explore how much the occurrence of disease A changes the probability of disease B occurrence and to verify the stability of the comorbidity pattern of diseases A and B, this study further calculated the lift of diseases A and B, as shown in **Table 5**. In terms of the comorbidity combination of schizophrenia, grade 3 hypertension (extremely high risk), and type 2 diabetes, the lift of {number of participants with grade 3 hypertension (extremely high risk) ->type 2 diabetes} and {type 2 diabetes ->grade 3 hypertension (extremely high risk)} was 3.361, indicating that the probability of having type 2 diabetes at the same time that participants with schizophrenia have grade 3 hypertension (extremely high risk) was 3.361 times greater than schizophrenia participants who did not have grade 3 hypertension (extremely high risk) but have type 2 diabetes. This means that schizophrenia participants with grade 3 hypertension (extremely high risk) increase schizophrenia participants with type 2 the incidence rate of diabetes, on the contrary, schizophrenia participants with type 2 diabetes also increased the incidence rate of grade 3 hypertension (extremely high risk). Considering the comprehensive analysis of support, confidence and lift, {grade 3 hypertension (extremely high risk) -> type 2 diabetes}, and {grade 3 hypertension (extremely high risk) -> type 2 diabetes, schizophrenia} suggests that these two comorbidity models were relatively stable.

For the comorbidities of schizophrenia, pulmonary infection and COVID-19 infection, {pulmonary infection->COVID-19 infection}, the lift was 3.514. In addition, for {COVID-19 infection->lung infection}, the lift was 3.524. For {pulmonary infection, schizophrenia->COVID-19 infection} and {COVID-19 infection->pulmonary infection, schizophrenia}, the lift was 3.514. For {schizophrenia, COVID-19 infection ->lung infection} and {lung infection->COVID-19 infection, schizophrenia}, the lift was 3.479.From The lift analysis of the comorbidity combination of schizophrenia, lung infection and COVID-19 infection revealed that, in the case of COVID-19 infection in schizophrenic participants, the probability of simultaneous lung infection was 3.479 times greater than that in schizophrenic participants without COVID-19 infection but with lung infection. The probability of COVID-19 infection in schizophrenic participants with lung infection was 3.514 times greater than that of COVID-19 infection without lung infection. According to the analysis results, the comorbidity patterns of schizophrenia, COVID-19 infection, and lung infection among inparticipants with schizophrenia were relatively stable, and schizophrenia participants with lung infection were more likely to suffer from COVID-19 infection.

In the comorbidity combination of schizophrenia, constipation, and acute nasopharyngitis[cold], the lift values for the associations {constipation->acute nasopharyngitis[cold]}, {schizophrenia, constipation->acute nasopharyngitis[cold]}, and {constipation->schizophrenia, acute nasopharyngitis [cold]} were all 1.779. In terms of support, confidence, and lift, among participants with schizophrenia, those who also have constipation were 1.779 times more likely to develop acute nasopharyngitis [cold] compared to schizophrenia participants without constipation but with acute nasopharyngitis [cold]. These findings indicate that schizophrenia participants with constipation are more likely to develop acute nasopharyngitis[cold].

In the comorbidity combination of schizophrenia, hypokalaemia, and acute nasopharyngitis[cold], the lift values for the associations {hypokalaemia->acute nasopharyngitis[cold]}, {acute nasopharyngitis [cold]-> hypokalaemia}, {schizophrenia, acute nasopharyngitis [cold]->hypokalaemia}, and {hypokalaemia->schizophrenia, acute nasopharyngitis [cold]} were all 1.318. The lift values for {hypokalaemia, schizophrenia->acute nasopharyngitis[cold]} and {schizophrenia, acute nasopharyngitis[cold]->hypokalaemia} were both 1.328. Based on the analysis of lift values, the comorbidity combination of hypokalaemia and acute nasopharyngitis [cold] among hospitalized participants with schizophrenia appears relatively stable.

## 4 Discussion

Participants with schizophrenia often present with both psychiatric and physiological disorders, requiring unique therapeutic considerations during treatment. Analyzing comorbidity characteristics and patterns can facilitate integrated interventions that address both mental and physical health aspects.

### 4.1 Sociodemographic factors and hospitalization characteristics significantly affect the comorbidity rate of schizophrenia participants

The comorbidity rate of hospitalized participants with schizophrenia is relatively high and shows significant age differences. The comorbidity rate is lower in the adolescent population, and higher in the middle-aged and elderly populations. As people with schizophrenia age, they not only face challenges related to their mental health status, but their physical function also gradually declines, leading to an increasing number of comorbidities. This trend is closely related to the physiological aging process and the negative effects of long-term psychiatric drug treatment, especially antipsychotic drugs. In addition to age factors, participants’ occupational status, marital status, length of hospital stay, number of hospitalizations, and medical insurance status have a significant impact on the number of comorbidities among hospitalized participants with schizophrenia. Unemployment and lack of spouses may mean less social support, unhealthy lifestyles, and decreased adherence to medical services, thereby exacerbating physical health risks. The chronic trend of schizophrenia itself and the complexity of mental illness and physical health determine the high frequency and long duration of hospitalization. In China, the type of medical insurance coverage is determined by occupational status, so urban and rural residents’ medical insurance plays an important role in the treatment of schizophrenia participants (27).

### 4.2 Comorbidity characteristics dominated by metabolic disorders, infections, and cardiovascular disease in schizophrenia participants

This study systematically reveals the complex comorbidity spectrum of hospitalized participants with schizophrenia, characterized by the widespread coexistence of schizophrenia with metabolic disorders, infections, and cardiovascular diseases, covering multiple systems. According to the classification code analysis of the ICD-10, schizophrenia participants have a high incidence of infectious diseases (J00-J06, U07, J95-J99), which may be due to the suppression of immune function caused by long-term medication, and the lack of self-protection ability caused by the disease itself also increases the risk of infectious diseases (28).

The incidence of metabolic cardiovascular disease clusters (E70-E90, I10-I15, E10-E14, I60-I69) is relatively high in schizophrenia participants. Long term use of antipsychotic drugs can lead to side effects such as weight gain and abnormal glucose and lipid metabolism. In addition, the cumulative effect of unhealthy lifestyle habits further increases the comorbidity risk of schizophrenia participants. In addition, participants with schizophrenia are at a higher risk of both delayed mental development (F70-F79) and immune system disorders (D80-D89). Furthermore, according to the ICD-10 code, it is confirmed that schizophrenia participants have comorbidity characteristics of infectious diseases, metabolic-cardiovascular disease, basic neurodevelopment and immune disease.

### 4.3 Schizophrenia’s four comorbidity patterns: pathological network mechanisms

Through association rule analysis, four relatively robust comorbidity patterns were identified among hospitalized individuals with schizophrenia. These patterns not only reveal the pathophysiological connections between diseases, but also point to key targets for precise intervention. The first comorbidity pattern is the metabolic-cardiovascular comorbidities cluster: schizophrenia, grade 3 hypertension (very high risk), type 2 diabetes mellitus, and showed a strong two-way correlation. Specifically, in individuals with schizophrenia, a synergistic deterioration mechanism is observed between grade 3 hypertension (very high risk) and type 2 diabetes mellitus. Insulin resistance accelerates vascular sclerosis, and the activation of renin angiotensin system further aggravates the glucose metabolism disorder r (29, 30). The second comorbidity pattern identified is the co-disease cluster of respiratory system infection: schizophrenia, COVID-19 infection, and pulmonary infections also have two-way correlation.COVID-19 infection often leads to secondary pulmonary infections due to its propensity to damage the respiratory mucosa, thereby compromising the respiratory tract’s defense mechanisms. Conversely, chronic pulmonary infections can increase the risk of COVID-19 exacerbation, potentially due to pre-existing lung inflammation and impaired respiratory function (31).

The third comorbidity pattern identified is the gut-respiratory symptom cluster, which includes schizophrenia, constipation, and acute nasopharyngitis[cold].A unidirectional high-confidence association exists between constipation and acute nasopharyngitis[cold], reflecting a cascade effect stemming from autonomic dysfunction(constipation ->acute nasopharyngitis [cold]). Specifically, anticholinergic medications, often used in the treatment of schizophrenia, can inhibit intestinal peristalsis, leading to constipation. This gastrointestinal disturbance can subsequently disrupt the gut microbiota balance. The resultant dysbiosis may further compromise the immune function of the respiratory mucosa, thereby increasing the risk of acute nasopharyngitis[cold] (32, 33). The fourth comorbidity pattern is electrolyte imbalance infection trigger cluster. Schizophrenia, hypokalemia, and acute nasopharyngitis[cold] also have stable bidirectional associations. Given these associations, routine monitoring of serum potassium levels is recommended for individuals with recurrent respiratory infections, as this may help in early detection and management of hypokalemia, thereby mitigating the risk of exacerbations and complications (34, 35).

The comorbidities of schizophrenia do not exist in isolation, but rather reinforce each other through specific pathological networks. Identifying these strong association patterns can shift clinical interventions from “single disease combination therapy” to “targeted blockade based on comorbidity networks”, providing a scientific pathway for improving overall patient prognosis. While this study has yielded valuable insights, it also has certain limitations. Specifically, the longitudinal comparison of sample data was insufficient, and there was an absence of direct contrasts between comorbidity patterns in schizophrenia patients and those in non-schizophrenia populations. In future research, it is necessary to further explore the changes and evolutionary trends of comorbidity patterns in participants with schizophrenia, as well as the differences in comorbidity patterns with non-schizophrenia participants, in order to make the treatment and care of schizophrenia more precise and improve the quality of life of participants.

## 5 Conclusion

This study employed an integrative approach combining statistical analysis and data mining techniques to investigate comorbidity features and interaction patterns among hospitalized participants with schizophrenia, from the perspectives of social demographic factors, disease characteristics, comorbidity features, and comorbidity pattern mining. These findings partially elucidate the associations between physical and mental disorders. As schizophrenia is a severe psychiatric condition, the analysis of schizophrenia comorbidity profiles provides valuable references for optimizing clinical diagnosis and patient health management.

## Data Availability

The datasets presented in this article are not readily available because this data involves patient privacy and, according to the relevant provisions of China’s Mental Health Law, shall not be publicly disclosed. Requests to access the datasets should be directed to LF, lifen_67@sina.com.

## References

[1] Lokkerbol J Adema D Graaf RD Have MT Smit F. Nonfatal burden of disease due to mental disorders in the Netherlands. Soc Psychiatry Psychiatr Epidemiol. (2013) 48:1591–9. doi: 10.1007/s00127-013-0660-8, PMID: 23397319

[2] Guo Z Lou L Wang Y Wang H Yao F Zhang R. A study on influencing factors of drug compliance in Henanserious mental disorder participants based on BPNN. J Zhengzhou Univ (Medical Sciences). (2020) 55:689–93. doi: 10.13705/j.issn.1671-6825.2019.08.146

[3] Liu Z. Characteristics, problems, and optimization paths of mental health service policies in the new era: an analysis framework based on policy concepts, objectives, and tools. Academics. (2023) 09:155–67. doi: 10.3969/j.issn.1002-1698.2023.09.016

[4] Yao F Zhang W Zhang R Liu C Wang H Guo Z. Management and treatment of severe mental disorders in Henan province of China. Chin Gen Pract. (2020) 23:2702–8.

[5] Xu X Mishra GD Jones M. Evidence on multimorbidity from definition to intervention: an overview of systematic reviews. Ageing Res Rev. (2017) 37:53–68. doi: 10.1016/j.arr.2017.05.003, PMID: 28511964

[6] WHO. Multimorbidity: technical series on safer primary care. Geneva: World Health Organization (2016).

[7] Dorrington SK Carr E Stevelink S Woodhead C Das-Munshi J Ashworth M. Multimorbidity and fit note receipt in working age adults with long-term health conditions. psychol Med. (2020) 52:1156–65. doi: 10.1017/S0033291720002937, PMID: 32895068

[8] Kugathasan P Wu H Gaughran F René EN Pritchard M Dobson R. Association of physical health multimorbidity with mortality in people with schizophrenia spectrum disorders: using a novel semantic search system that captures physical diseases in electronic patient records. Schizophr Res. (2020) 216:408–15. doi: 10.1016/j.schres.2019.10.061, PMID: 31787481

[9] Bendayan R Kraljevic Z Shaari S Das-Munshi J Leipold L Chaturvedi J. Mapping multimorbidity in individuals with schizophrenia and bipolar disorders: evidence from the south London and Maudsley NHS foundation trust biomedical research centre (SLAM BRC) case register. BMJ Open. (2022) 12:e054414. doi: 10.1136/bmjopen-2021-054414, PMID: 35074819 PMC8788233

[10] Halstead S Cao C Hognason MG Ebdrup BH Pillinger T Mccutcheon RA. Prevalence of multimorbidity in people with and without severe mental illness: a systematic review and meta-analysis. Lancet Psychiatry. (2024) 6):11. doi: 10.1016/S2215-0366(24)00091-9, PMID: 38642560

[11] WHO. The world health report 2001-Mental Health: New Understanding, New Hope. Available online at: https://www.who.int/whr/2001/en/ (Accessed October 09, 2024).

[12] Oliveira FES Trezena S Martelli DRB Oliveira MCL Colosimo EA Júnior HM. The association between schizophrenia and increased Covid-19 mortality in a cohort of over 2 million people in Brazil. Braz J Psychiatry. (2024) 46:e20243540. doi: 10.47626/1516-4446-2024-3540, PMID: 38635950 PMC11559914

[13] Oliveira FES Martelli-Júnior H Colosimo EA ACSe S CS D LO D. Vaccine protection against COVID-19-related death in participants with schizophrenia: A retrospective cohort study. Braz J Psychiatry. (2022) 0:1–17. doi: 10.47626/1516-4446-2024-4024 PMC12815387 41485244

[14] Andrew S Daisy F. Protecting physical health in people with mental illness. Lancet Psychiatry. (2019) 6:889–90. doi: 10.1016/S2215-0366(19)30393-1, PMID: 31631866

[15] Lawrence D Kisely S Pais J. The epidemiology of excess mortality in people with mental illness. Can J Psychiatry. (2010) 55:752–60.3. doi: 10.1177/070674371005501202, PMID: 21172095

[16] Reilly S Olier I Planner C Doran T Reeves D Ashcroft DM. Inequalities in physical comorbidity: a longitudinal comparative cohort study of people with severe mental illness in the UK. BMJ Open. (2015) 5:e009010. doi: 10.1136/bmjopen-2015-009010, PMID: 26671955 PMC4679912

[17] Woodhead C Ashworth M Schofield P Henderson M. Patterns of physical co/multimorbidity among participants with serious mental illness: a London borough-based cross-sectional study. BMC Family Practice. (2014) 15:117. doi: 10.1186/1471-2296-15-117, PMID: 24919453 PMC4062514

[18] Mccreadie RG. Diet, smoking and cardiovascular risk in people with schizophrenia: Descriptive study. Br J Psychiatry. (2019) 183:534–9. doi: 10.1192/bjp.183.6.534, PMID: 14645025

[19] Carrà G Scioli R Monti MC Marinoni A. Severity profiles of substance-abusing participants in Italian community addiction facilities: influence of psychiatric concurrent disorders. Eur Addict Res. (2022) 12:96–101. doi: 10.1159/000090429, PMID: 16543745

[20] Carrà G Johnson S Crocamo C Angermeyer MC Brugha T Azorin JM. Psychosocial functioning, quality of life and clinical correlates of comorbid alcohol and drug dependence syndromes in people with schizophrenia across Europe. Psychiatry Res. (2016) 239:301–7. doi: 10.1016/j.psychres.2016.03.038, PMID: 27046394

[21] Plana-Ripoll O Pedersen CB Agerbo E Holtz Y Erlangsen A Canudas-Romo V. A comprehensive analysis of mortality-related health metrics associated with mental disorders: a nationwide, register-based cohort study. Lancet. (2019) 394:1827–35. doi: 10.1016/s0140-6736(19)32316-5, PMID: 31668728

[22] Weye N Momen NC Whiteford HA Christensen MK Iburg KM Santomauro DF. The contribution of general medical conditions to the nonfatal burden of mental disorders: register-based cohort study in Denmark. (Cambridge, England: Cambridge University Press) 8. (2022). doi: 10.1192/bjo.2022.583, PMID: 36205020 PMC9634585

[23] Szoke A Pignon B Godin O Ferchiou A Tamouza R Leboyer M. Multimorbidity and the aetiology of schizophrenia. Curr Psychiatry Rep. (2024) 26:253–63. doi: 10.1007/s11920-024-01500-9, PMID: 38625632

[24] Correll CU Solmi M Veronese N Bortolato B Rosson S Santonastaso P. Prevalence, incidence and mortality from cardiovascular disease in participants with pooled and specific severe mental illness: a large-scale meta-analysis of 3, 211, 768 participants and 113, 383, 368 controls. World Psychiatry. (2017) 16:163–80. doi: 10.1002/wps.20420, PMID: 28498599 PMC5428179

[25] Bhalla IP Stefanovics EA Rosenheck RA. Mental health multimorbidity and poor quality of life in participants with schizophrenia. Schizophr Res. (2018) 201:39–45. doi: 10.1016/j.schres.2018.04.035, PMID: 29709490

[26] Yu J. A Study on Multimorbidity Patterns and the Cost of Inparticipants in Jilin Province. Jilin, China: Jilin University (2022). doi: 10.27162/d.cnki.gjlin.2022.007177

[27] Galderisi S De Hert M Del Prato S Fagiolini A Gorwood P Leucht S. Identification and management of cardiometabolic risk in subjects with schizophrenia spectrum disorders: A Delphi expert consensus study. Eur Psychiatry. (2020) 64:e7. doi: 10.1192/j.eurpsy.2020.115, PMID: 33413701 PMC8057390

[28] Bartoli F Crocamo C Clerici M Carrà G. Second-generation antipsychotics and adiponectin levels in schizophrenia: A comparative meta-analysis. Eur Neuropsychopharmacol. (2015) 25:1767–74. doi: 10.1016/j.euroneuro.2015.06.011, PMID: 26164075

[29] Nielsen J Skadhede S Correll CU. Antipsychotics associated with the development of type 2 diabetes in antipsychotic-nave schizophrenia patients. Neuropsychopharmacology. (2010) 35:1997–2004. doi: 10.1038/npp.2010.78, PMID: 20520598 PMC3055629

[30] Sharma A Basera DS Suri V Singh SM. A study of hypertension and related biophysical and health-related lifestyle behaviors in patients suffering from schizophrenia. Ann Neurosciences. (2024) 31:28–35. doi: 10.1177/09727531231158451, PMID: 38584984 PMC10996874

[31] Bravve L Zakharova N. COVID-19-associated schizophrenia-like psychosis during the COVID-19 pandemic. Eur Psychiatry. (2023) 66:1. doi: 10.1192/j.eurpsy.2023.1689

[32] Luo Q An M Wu Y Wang J Mao Y Zhang L. Genetic overlap between schizophrenia and constipation: insights from a genome-wide association study in a European population. Ann Gen Psychiatry. (2025) 24(1):11. doi: 10.1186/s12991-025-00551-3, PMID: 40033405 PMC11877780

[33] Gopal S Vijapurkar U Lim P Morozova M Eerdekens M Hough D. A 52-week open-label study of the safety and tolerability of paliperidone palmitate in patients with schizophrenia. J Psychopharmacol. (2011) 25:685–97. doi: 10.1177/0269881110372817, PMID: 20615933

[34] Jun DW Kim SH Kim K Jang WY Ks N Kim H. Hyponatremia in schizophrenia after water intoxification: a potential cause of rhabdomyolysis. Korean J Internal Med. (2000) 59:335–8.

[35] Azad MC Shoesmith WD Al Mamun M Abdullah AF Naing DK Phanindranath M. Cardiovascular diseases among patients with schizophrenia. Asian J Psychiatr. (2016) (19):28–36. doi: 10.1016/j.ajp.2015.11.012, PMID: 26957335

